# Time-programmable coloration via 3D metastructures for optical encryption

**DOI:** 10.1038/s41377-026-02202-y

**Published:** 2026-02-19

**Authors:** Ming-Ze Zhao, Zhi-Yong Hu, Yi-Han Tao, Ze-Xin Zhou, Li-Jun He, Zhen-Nan Tian, Xue-Qing Liu, Qi-Dai Chen, Din Ping Tsai

**Affiliations:** 1 State Key Laboratory of Integrated Optoelectronics, College of Electronic Science and Engineering, Jilin University, 2699 Qianjin Street, Changchun, 130012 China; 2 Department of Electrical Engineering and State Key Laboratory of Optical Quantum Materials, City University of Hong Kong, Hong Kong, 999077 China; 3 College of Physics, Jilin University, Changchun, 130012 China; 4 State Key Laboratory of Terahertz and Millimeter Waves, City University of Hong Kong, Hong Kong, 999077 China

**Keywords:** Metamaterials, Lithography, Optical data storage

## Abstract

Optical information encryption technology, with its advantages such as high-dimensional encryption characteristics and resistance to quantum computing decryption, has demonstrated unique application value in fields like military, communication, and commercial confidential information protection. However, current optical encryption technologies still face challenges such as limited information capacity and security protection bottlenecks due to discrete state switching mechanisms, as well as potential risks of information residual leakage. Here, we propose a continuously tunable time-programmable coloration encryption strategy enabled by three-dimensional metastructures, featuring wide color gamut spectral continuous tuning through the control of the environmental refractive index. Moreover, by innovatively inducing irreversible collapse of nanopillars through capillary forces, the encryption carrier is endowed with physical self-destruction characteristics, thereby enabling “burn after reading” of the encrypted information. As a proof of concept, we demonstrated time-programmable information encryption and self-destruction after reading within a single device, enabled by continuous spectral modulation across the visible wavelength range. This technology provides an innovative solution for dynamic response in information encryption and secure information destruction, showing significant application potential in high-security scenarios such as military confidential transmissions and high-end commercial anti-counterfeiting.

## Abstract (web summary)

A continuously tunable time-programmable 3D metastructure coloration encryption approach with wide-gamut spectral control via refractive-index modulation and capillary-driven self-destruction is presented for high-capacity secure information encryption.

## Graphical abstract

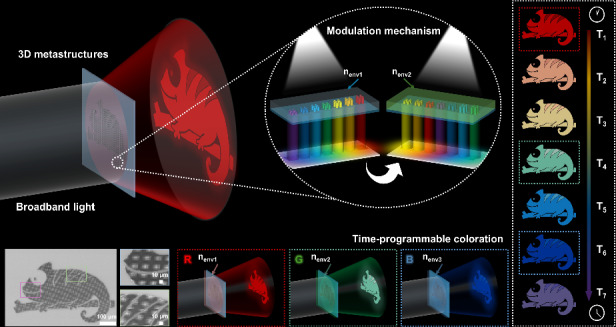

## Introduction

With the rapid development of the information age, information encryption technology has become a core support for high-security fields such as military communications, medical privacy, and commercial confidentiality^1^. Although traditional electronic encryption technologies (such as symmetric and asymmetric encryption) have matured, their reliance on mathematical algorithms renders them vulnerable to quantum computing attacks^2,3^, while loose key management further exacerbates system vulnerabilities^4^. In contrast to electronic encryption, optical encryption technology, with its physical characteristics independent of mathematical dependencies, avoids the risk of quantum decryption at the fundamental level^5^. Moreover, it enables higher-dimensional information encoding through the combined control of multiple physical parameters and degrees of freedom, significantly enhancing resistance to cracking^6–8^. Optical encryption, with its high security, high-dimensional encryption, and high design flexibility, has become an important path to overcoming the bottlenecks of traditional information encryption technologies^9–16^.

Micro-nano structures, due to their ability to precisely manipulate light waves, are considered among the ideal carriers for optical encryption because of their advantages such as no need for dyes, high stability, and environmental friendliness^17–22^. These structures not only enable high-resolution static color representation but also possess unique multi-physical field response potential in dynamic information encryption^23–26^. However, existing optical dynamic encryption strategies typically trigger a binary color switch (such as “red/blue” or “visible/invisible”) through a single physical parameter (e.g., temperature, humidity, or polarization)^27–29^. This “binary switching” mode, owing to its discrete-state nature, is difficult to achieve time-programmable continuous encoding, thereby limiting information capacity and making it vulnerable to brute-force attacks based on multi-parameter analysis. Additionally, another bottleneck in optical dynamic encryption technology is the risk of secondary leakage after information retrieval^30,31^. If the information carrier does not immediately destroy the data after receiving it, confidential information may still be exposed later. Current solutions often rely on chemical reactions or external energy inputs^32,33^, which suffer from issues such as limited substrate adaptability, strong environmental dependence, chemical residue contamination, and biological toxicity. Therefore, developing encryption technologies that integrate continuous color gamut control, multi-dimensional dynamic coding, and self-destruction functions has become a key challenge in breaking through the limitations of traditional binary states and building high-security information carriers.

To address the aforementioned challenges, this study proposes a far-field time-programmable coloration strategy enabled by 3D metastructures for high-security optical encryption. By precisely designing the geometric parameters of the metastructural units, the spectral response characteristics can be accurately controlled. Moreover, through controlled modulation of the environmental refractive index, the colors appear sequentially in a preset temporal order, thereby achieving time-programmable structural coloration. Furthermore, irreversible deformation or collapse of the metastructures induced by capillary forces can trigger the self-destruction of the encrypted information, forming a green and physically secure destruction mechanism. This time-programmable and continuously tunable coloration technology overcomes the bottlenecks of traditional optical encryption technologies constrained by discrete-state switching, thereby enabling time-programmable information encryption within a single physical carrier. As a proof of concept, this study demonstrates precisely tunable coloration spanning from red to violet across the visible spectrum and achieves the implementation of time-programmable information encryption on a single metastructure array, along with information self-destruction after reading. By integrating refractive-index-mediated programmable coloration with capillary-force-driven information self-destruction, this technical architecture provides a dual security mechanism of dynamic programmability and physical destruction, offering significant potential for applications in high-risk scenarios.

## Results

### Modulation mechanism of time-programmable coloration

**Fig. 1:**
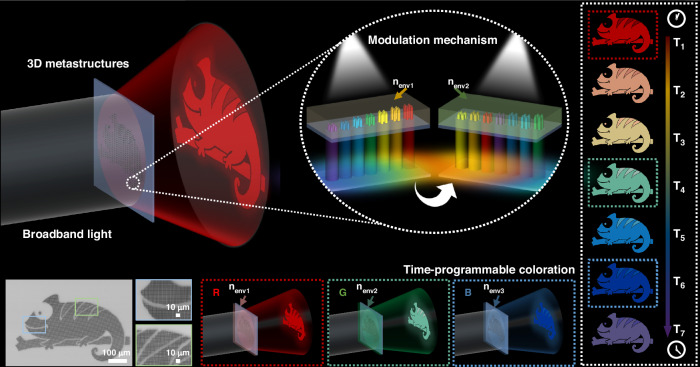
Working principle and experimental characterization of time-programmable coloration. Under broadband white light illumination, the designed 3D metastructures precisely modulate the optical field to generate predefined colors and patterns in the far field. The enlarged inset illustrates the modulation mechanism of the structural colors produced by the 3D metastructures, where n_env1_, n_env2_ and n_env3_ denote the environmental refractive indices corresponding to different device working environments. Morphological characterization of the chameleon-inspired patterned 3D metastructures is shown with scanning electron microscopy (SEM) images of the complete pattern and a magnified local region (bottom left). Time-programmable continuous color tuning achieved under varying environmental refractive indices is illustrated on the right, where T₁–T₇ represent the corresponding programmable time points. The bottom insets depict representative R/G/B output states under three distinct refractive index conditions

As illustrated in Fig. 1, when illuminated by broadband white light, the designed 3D metastructures allow only the light at the designed wavelength to be transmitted at a small angle and form a clear image in the far field, while other wavelengths are scattered at larger angles and thus suppressed, resulting in a monochromatic structural coloration effect. The wide-gamut coloration across the visible spectrum originates from the combined effects of Mie resonances and diffraction, with Mie resonances playing the dominant role and being strongly dependent on the geometric dimensions of the structures. Therefore, the coloration technology enabled by 3D metastructures requires precise design and fabrication of parameters such as diameter, period, and height of the structures^34,35^. Compared with traditional micro-nanofabrication techniques (such as ultraviolet lithography, electron/ion beam etching, and nanoimprinting), femtosecond laser 3D printing (FsL-3DP) based on two-photon polymerization (TPP) offers significant advantages. Owing to its nonlinear two-photon absorption characteristics and high energy threshold, FsL-3DP overcomes the planar limitations of conventional methods and enables subwavelength-scale fabrication resolution in 3D space, making it an ideal solution for realizing the designed 3D meta-devices^36–40^. As illustrated in Supplementary Fig. S1, a near-infrared FsL-3DP system was employed for the precise fabrication of patterned 3D metastructures (the dynamic fabrication process of the microstructures can be viewed in Supplementary Movie 1).

It is worth noting that the spectral and color presentation of the structural colors is usually inevitably influenced by factors such as the numerical aperture (NA) of the objective lens in the imaging system and the tilt angle of the sample. To evaluate the NA dependence, transmission spectra under different NA conditions were simulated (Supplementary Fig. S2). The results show that when the objective lens NA varies from 0.1 to 0.6, the main coloration wavelength of the structural patterns remains stable, with only minimal changes in the overall spectral shape. This conclusion is further supported by experimental results, where consistent structural coloration patterns were observed without significant color shifts when using objective lenses with different NA values (0.1–0.55) (Supplementary Fig. S3), in good agreement with the simulation. In addition, when the sample tilt angle deviates less than 15° from the normal direction, the main peak position of the transmission spectrum also remains largely unchanged (Supplementary Fig. S4). This excellent stability can be attributed to the weak angular dispersion characteristics of the designed metastructures, effectively reducing the system’s sensitivity to variations in the incident and collection angles.

Moreover, the coloration of 3D metastructures is not only determined by geometric parameters but also strongly dependent on the refractive index of the working environment. Building upon this foundation, dynamic modulation of the environmental refractive index enables time-programmable and continuously tunable coloration of the metastructure’s transmission spectra. By altering the refractive index contrast between the metastructures and the surrounding medium through external stimuli, the effective optical path length and phase distribution can be reconfigured, resulting in continuous yet programmable shifts of the transmission spectrum. This approach allows the structural colors to evolve along the time axis in a controlled and continuous manner. Simulation results (Supplementary Fig. S5) demonstrate that the transmission spectra of the same structure exhibit systematic shifts under different environmental refractive indices, confirming the feasibility of refractive-index-mediated time-programmable continuous coloration. This mechanism not only imparts time-programmable response characteristics with continuous tunability to structural color but also provides a new paradigm for information encryption and optical security, offering enhanced flexibility and robustness against potential attacks.

### Performance evaluation of as-fabricated metastructure colors

**Fig. 2:**
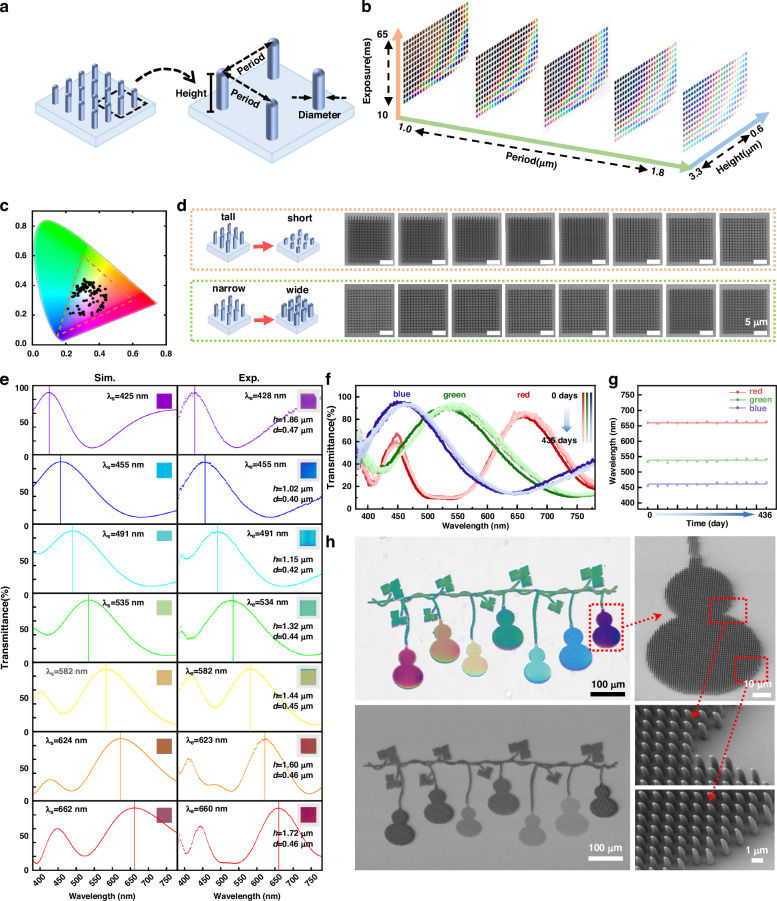
Performance evaluation of as-fabricated metastructure colors. **a** Schematic illustration of the characteristic parameters of the metastructures. **b** 3D transmission palette of metastructures with varying heights, periods, and exposure times. **c** CIE 1931 chromaticity diagram of structural colors at a period of 1.2 μm. **d** Schematic diagrams (left) and SEM images (right) illustrating the control of height (top) and diameter (bottom) in 3D metastructures. **e** Simulated (left) and experimental (right) normalized transmission spectra of monochromatic metastructure arrays, with insets showing the corresponding perceived colors. All structures share a period of 1.2 μm, and the labels below each inset denote the structure height h and diameter d. **f** Long-term transmission spectra of RGB metastructures, where color depth (five levels) indicates the progression of time. **g** Long-term statistical analysis of the peak position variations of the transmission spectra shown in (**f**). **h** Full-color 3D-printed structural color patterns. Transmission optical microscopy image (upper left) and panoramic SEM image (lower left); complete SEM image of the purple gourd-shaped structure (upper right) and corresponding magnified SEM view (lower right)

The coloration properties of structural colors are closely correlated with the height, diameter, and period of the metastructures (Fig. 2a). To systematically investigate the influence of these parameters on color modulation, a large-area 3D structural color palette was designed and fabricated. The diameter of the metastructures was controlled by adjusting the point exposure time in the FsL-3DP process. During the fabrication of the palette, the exposure time was varied from 10 to 65 ms (in 5 ms increments), the structure height ranged from 0.6 to 3.3 μm (in 0.1 μm steps), and the period ranged from 1.0 to 1.8 μm (in 0.2 μm steps), with a constant writing power of 15 mW. Observations under transmission optical microscopy (Fig. 2b) reveal that the printed structural colors exhibit excellent color gamut coverage. Notably, the color brightness correlates with the structural period, whereas the dominant hue is primarily governed by the structural height and diameter, with a stronger dependence on height (Detailed simulation results are provided in Supplementary Fig. S6). Figure 2c presents the CIE 1931 chromaticity diagram of structural colors with a fixed period of 1.2 μm, indicating a wide color gamut and confirming the outstanding color-rendering performance of the system. Furthermore, scanning electron microscopy (SEM) images of the microstructures in the 1.2 μm period palette, featuring gradients in height (1.3–0.6 μm) and diameter (corresponding to exposure times from 65 to 35 ms), clearly demonstrate the precise 3D control capability of the FsL-3DP technique (Fig. 2d). Based on these results, rainbow colors printing was successfully achieved, and the transmission spectra of monochromatic structural arrays corresponding to seven different colors were simulated and experimentally measured (Fig. 2e), showing good agreement between simulations and experiments. These results verify that the structural color can be precisely designed and modulated. The images and spectra of the metastructures were collected using the system shown in Supplementary Fig. S7.

Long-term stability and photostability are among the core metrics for evaluating the performance of color output devices. To systematically assess the long-term stability of structural colors, a stability tracking experiment spanning over 430 days was conducted. The evolution of the transmission spectra of RGB micro/nanostructures over the testing period (Fig. 2f), as well as the dynamic variations in the spectral peak positions (Fig. 2g), showed that the spectral curves at different time points exhibited high overlap, with fluctuations of the characteristic peak positions consistently controlled within ±5 nm, thereby fully confirming the excellent long-term stability of the structural colors. To evaluate photostability, a comparative photobleaching experiment between the structural colors and conventional dyes was designed. Under focused laser irradiation at 410 nm with a power of 8.54 mW for 3 h, both the structural color samples and Rhodamine B dye samples were tested. During the irradiation process, the fluorescence intensity of the traditional dyes decayed significantly, whereas the optical properties of the structural color samples remained stable (detailed comparative statistics are provided in Supplementary Fig. S8). This pronounced difference is attributed to the coloration mechanisms: unlike dyes, whose coloration relies on molecular structures, the physical coloration mechanism based on nanostructures effectively avoids photobleaching effects^41,42^. These results, evaluated from the perspectives of long-term stability and photostability, demonstrate that structural color technology exhibits superior durability compared to conventional dyes, offering unique advantages for applications requiring sustained color retention, such as anti-counterfeiting and information encryption.

In addition to a wide color gamut and excellent long-term stability and photostability, the practical application of structural colors also requires the capability to fabricate complex patterns. In this study, complex metastructured patterns were successfully printed by precisely programming the motion commands of the displacement stage and galvanometer in the FsL-3DP system. For monochromatic and multicolored patterns, a monochrome emblem of Jilin University and an RGB tricolor hot air balloon structure were successfully fabricated, respectively (Supplementary Fig. S9). To further demonstrate the capability of printing full-color complex patterns, Fig. 2h presents a structural color pattern featuring seven distinct colors, where the green design utilizes a higher structure than the rainbow one to obtain improved color saturation. The localized SEM images of the purple gourd-shaped structure in different regions reveal a uniform surface morphology, confirming the processing stability of the FsL-3DP technique. In summary, the 3D metastructure colors exhibit outstanding coloration performance, stability, and designability, demonstrating strong practical potential for applications requiring high security and durability, such as anti-counterfeiting labels, information storage, and encryption.

### Structural color-based anti-counterfeiting labels and information storage

**Fig. 3:**
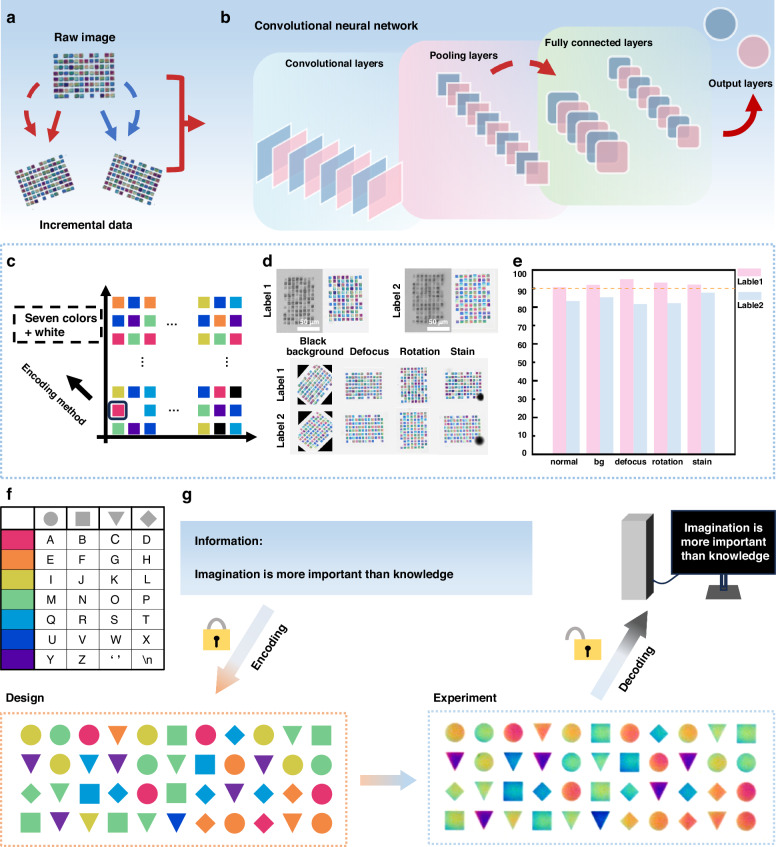
Structural color-based anti-counterfeiting labels and information storage. **a** Schematic illustration of label data augmentation. **b** Schematic diagram of the convolutional neural network (CNN) architecture. **c** Schematic diagram of label encoding. **d** Transmission optical microscopy and SEM images of labels under normal conditions (top); transmission optical microscopy images of labels under different conditions (bottom), including black background, defocus, rotation, and stain. **e** Recognition results for unknown labels. **f** Schematic illustration of structural color information encoding rules. **g** Schematic diagram of encoding the designated information “Imagination is more important than knowledge” into a structural color matrix based on the encoding rules, along with the transmission optical microscopy image of the fabricated structural color matrix and its decoding illustration

Leveraging the superior performance of structural colors in precise color modulation, long-term stability, and structural designability, their application potential in micro/nano-optical anti-counterfeiting and information storage has been further explored. For anti-counterfeiting purposes, two structural color anti-counterfeiting label templates were designed (Supplementary Fig. S10), and batch fabrication of structural color label arrays with submicron morphological uniformity was achieved using FsL-3DP technology (Supplementary Fig. S11). The fabricated labels exhibited high uniformity in both macroscopic morphology and coloration characteristics, demonstrating the excellent stability and reproducibility of the manufacturing process and providing a solid foundation for the practical deployment of large-scale anti-counterfeiting applications. To enable efficient and accurate recognition of the anti-counterfeiting labels, a convolutional neural network (CNN)-based recognition model tailored for structural color labels was developed. Considering the difficulty in constructing effective negative sample sets in real-world scenarios where counterfeit labels lack standardized formats, only positive samples (Label 1) were used to build the initial training dataset, thereby realistically simulating practical application conditions. To address the challenges of model training under limited sample conditions, image data augmentation strategies were employed, expanding the original dataset tenfold through geometric transformations such as image rotation and scaling (Fig. 3a), significantly enhancing the model’s adaptability and robustness to image features. Figure 3b shows the architecture of the constructed CNN model. In addition, Fig. 3c illustrates the coding scheme for the anti-counterfeiting labels, where each label consists of 8 × 12 color units, with each unit capable of displaying eight different colors (including rainbow colors and white). The spatial arrangement of different colors generates a vast number of possible combinations, endowing the coding structure with high information entropy and design flexibility, thus significantly enhancing the security and forgery resistance of the labels. To verify the practical anti-counterfeiting performance of the system, a testing set containing five scenarios (normal, black background, defocus, rotation, and stain) was constructed, as shown in Fig. 3d. Experimental results (Fig. 3e) show that when the confidence threshold was set to 90, the CNN achieved an accuracy of 99.4% over 530 recognition attempts, with true positive rate (TPR), false positive rate (FPR), true negative rate (TNR), and false negative rate (FNR) of 99.25%, 0%, 100%, and 0.75%, respectively. These results confirm the robustness of the structural color-based anti-counterfeiting system under complex environmental conditions. Benefiting from its submicron-scale resolution (Supplementary Fig. S12, minimum color unit <1 μm), the structural color anti-counterfeiting design offers high replication cost and difficulty, suitable for machine-fingerprint authentication of high-value devices.

In the field of information storage, the FsL-3DP technique was employed to fabricate microscale barcodes and QR codes for information encoding and retrieval within limited spaces (Supplementary Fig. S13), demonstrating the potential of structured patterns for high-density storage. Building upon this, a more scalable and readable structural color encoding system was further developed. As illustrated in Fig. 3f, by combining seven distinct structural colors with four distinct geometric configurations, an information mapping library containing 28 independent encoding units was established to represent English letters, spaces, and line breaks. To verify the application potential of this system in information storage, a conceptual demonstration was conducted by encoding the famous quote by Einstein, “Imagination is more important than knowledge.” A total of 44 characters were encoded into an 11 × 4 structural color matrix and fabricated as a microscale pattern using FsL-3DP (Fig. 3g), with a center-to-center spacing of 120 μm between encoding units, achieving an information density of up to 5.56 × 10⁸ bits/m². Furthermore, the encoded array can be accurately recognized and decoded using computer vision algorithms combined with decoding rules. The intrinsic stability of structural colors facilitates long-term information preservation. The effective information capacity of this storage scheme depends on the receiver’s minimum discernible color-difference threshold, fabrication accuracy, structural tolerance, minimum pixel size, and the dimensionality of the encoding. Notably, the minimum pixel in our scheme can be realized by a single nanopillar, which offers a higher upper bound on capacity. With further improvements in FsL-3DP fabrication resolution and expansions in the encoding dimensions—such as incorporating richer structural color features or multilayer encoding strategies—the information carrying capacity per unit area of the system can be significantly enhanced, providing a novel pathway toward the development of next-generation high-density, visual, and sustainable information storage technologies.

### Time-programmable structural colors for optical information encryption

The spectral response of the metastructures is highly sensitive to the refractive index of the surrounding medium, thereby enabling time-programmable and continuously tunable coloration through refractive index modulation. Liquid tuning, which offers advantages such as cleanliness, low cost, and environmental friendliness, serves as an effective approach for refractive index modulation^43,44^. Specifically, by introducing liquids with tailored refractive indices, the refractive index contrast between the nanostructures and their environment can be adjusted, providing precise control over the resulting structural coloration. Moreover, beyond spectral tunability, the infiltration of liquids also induces a mechanical response at the structural level, further enriching the multi-physics functionality of the system. During the evaporation process, the metastructures are subjected to a competing interplay between lateral capillary forces (${F}_{C}$) and structural support forces (${F}_{S}$) (Supplementary Fig. S14), which can be respectively expressed as^45–48^

1 $${F}_{C}\approx \frac{\gamma {hw}\cos \theta }{d}$$

2 $${F}_{S}\approx E\frac{{w}^{4}d}{{h}^{3}}$$

**Fig. 4:**
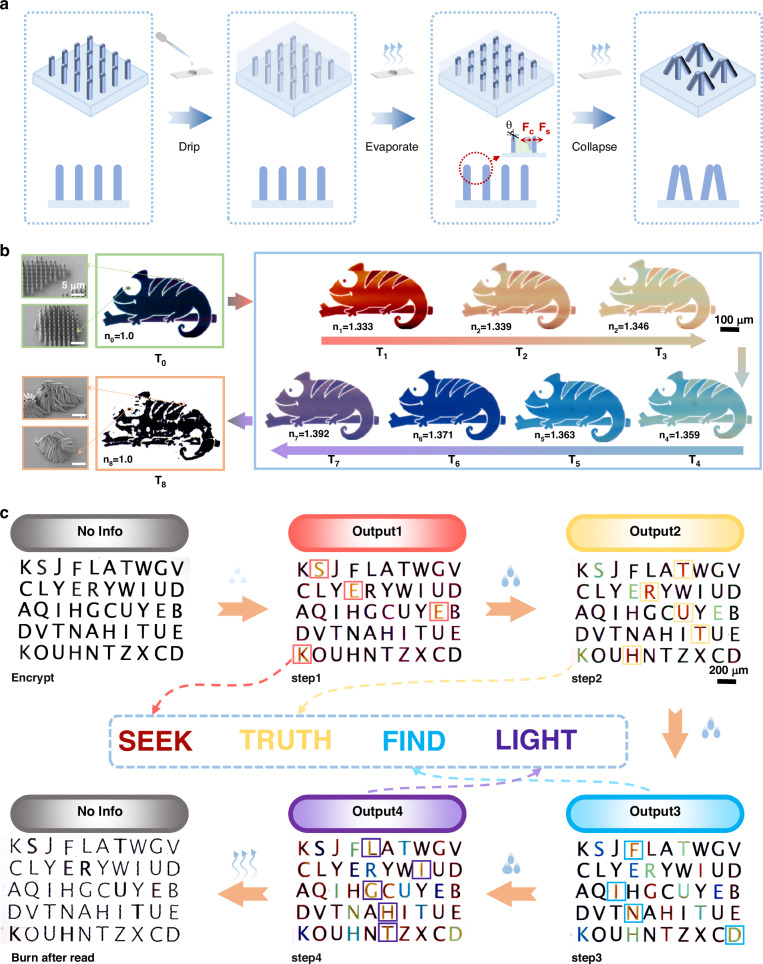
Time-programmable structural colors for optical information encryption. **a** Schematic illustration of the self-erasing process. **b** Optical microscopy images showing the time-programmable coloration and self-destruction process of the chameleon pattern at different time points (T₀–T₈). T₀ represents the initial state in air; T₁–T₇ correspond to the sequential programmable color transition from red to purple; T₈ denotes the final state after structural collapse. n_0_–n_8_ denote the environmental refractive indices of the structure at the corresponding time points. Insets on the left show the corresponding SEM images at T₀ and T₈. **c** Transmission optical microscopy images of time-programmable encrypted information at different decryption steps, including the initial state, decryption results of the first to fourth layers (Output1-4), and the final state after structural self-destruction

Among them, $\gamma$ denotes the surface tension between the liquid and the structure, $h$ and $w$ represent the height and diameter of the metastructures, respectively, $\theta$ is the contact angle between the liquid and the metastructures, $d$ is the period of the structures, and $E$ is the Young’s modulus of the structural material. As indicated by Eqs. (1) and (2), to enable automatic collapse of the structures during liquid evaporation and thereby accomplish secure information destruction, the metastructures must possess appropriate height and diameter, together with a relatively small period, so that the deformation threshold can be reached under capillary forces. However, if the designed height is excessively large or if the diameter and period are too small, the mechanical stability of the structures would be significantly reduced, making them vulnerable to unintended damage from liquid flow or diffusion, which could cause premature collapse or unwanted color distortion. Therefore, to simultaneously ensure reliable time-programmable coloration and irreversible collapse during the evaporation stage, the geometric parameters of the metastructures must be carefully optimized to balance optical performance with mechanical response (a detailed schematic is provided in Fig. 4a). This self-destructive mechanism operates without external energy input, featuring low energy consumption and environmental friendliness, thereby offering an efficient and sustainable pathway for green, secure information encryption and destruction.

Figure 4b illustrates the time-programmable coloration and self-destruction process of a chameleon pattern constructed based on this mechanism. In air, the pattern appears black, with SEM characterization confirming the orderly arrangement of the structures. Upon the introduction of deionized water (*n* = 1.33), the pattern instantly turns red. Subsequently, by gradually increasing the concentration of glycerol (*n* = 1.475) to create a continuous refractive index gradient, sequential and programmable modulation of the structural coloration is achieved. As time progresses, the pattern color transitions in a continuous and controllable manner from orange (*n* = 1.339), yellow (*n* = 1.346), green (*n* = 1.359), cyan (*n* = 1.363), and blue (*n* = 1.371) to purple (*n* = 1.392), thus realizing a full-spectrum programmable transition from red to purple across the entire visible range (as shown in Supplementary Movie 2). It is evident that an average change in refractive index of 0.010 already produces a discernible color difference, and a change of 0.059 yields a full transition from red to purple, indicating that the spectral response of the metastructures is highly sensitive to the refractive index of the surrounding medium. Finally, with the evaporation of the liquid, the pattern color disappears and the structures collapse. Transmission optical microscopy and SEM imaging confirm that the metastructures undergo irreversible deformation under capillary forces, thereby completing the secure self-destruction of the information.

The aforementioned time-programmable and continuously tunable coloration capability enables information to be dynamically encoded within a single physical structure, while the self-destruction mechanism provides a secure “burn after reading” functionality. Thus, this time-programmable structural coloration strategy offers an ideal pathway for constructing high-security optical information encryption systems. As a proof of concept, an encrypted structure containing four pieces of encoded information was designed (the geometric parameters design and detailed theoretical analysis are provided in Supplementary Fig. S15). Figure 4c presents the complete decryption and self-destruction process. In the initial state, a random array of letters appears uniformly black, and the encrypted information cannot be distinguished using transmission/reflection optical microscopy, dark-field microscopy, fluorescence microscopy, or SEM (Supplementary Fig. S16). Upon the introduction of deionized water, the first encrypted message, “SEEK,” immediately appears due to the change in environmental refractive index. Subsequently, as a refractive index gradient is established by the diffusion of glycerol, the following messages “TRUTH,” “FIND,” and “LIGHT” sequentially emerge, realizing a time-programmable decryption process (Supplementary Movie 3). Notably, the initial response is nearly synchronous with liquid coverage, whereas the emergence speed of subsequent messages is dictated by the diffusion rate of glycerol in water. Under current experimental conditions, the full decryption process takes ~125 s, but the response time could theoretically be reduced to the millisecond scale by employing liquids with faster diffusion rates. After all information is revealed, the system naturally enters the collapse phase during liquid evaporation, where capillary forces induce irreversible deformation of the nanostructures, achieving automatic and secure information erasure. Follow-up tests confirmed that even after liquid reintroduction, no color change or patterns reappeared, unequivocally demonstrating the one-time readability and complete destruction of the system (detailed in Supplementary Movie 4). Although only four messages are demonstrated here, the encoding capacity can be further expanded in practice, with the upper limit primarily determined by the receiver’s ability to discriminate color differences. Furthermore, introducing a combined transmission–reflection channel within the same device^49,50^, or superimposing orthogonal degrees of freedom such as polarization and incidence angle^51,52^, can significantly enhance the effective information capacity without increasing spatial multiplexing. This study therefore establishes a time-programmable coloration encryption strategy integrating programmable encoding, sequential decryption, and secure self-destruction, providing a novel pathway toward next-generation high-security optical encryption technologies.

## Discussion

In summary, this study proposes a high-security optical information encryption strategy based on 3D metastructures, featuring time-programmable coloration and secure self-destruction capabilities. Using FsL-3DP technology, precise and controllable fabrication of subwavelength-scale photonic structures was achieved. The resulting coloration exhibits a wide gamut, high spatial resolution, and excellent long-term stability and photostability. Under refractive index modulation, time-programmable and continuously tunable spectral responses were realized, enabling programmable information encryption within a single physical structure. Furthermore, a capillary-force-driven irreversible collapse mechanism was introduced to physically erase the information after reading, establishing a secure “burn after reading” encryption pathway. A proof-of-concept demonstration showcased the sequential emergence of four programmable encrypted messages under a refractive index gradient, followed by irreversible destruction upon solvent evaporation. This work not only expands the design freedom of optical encryption systems but also provides new insights into the development of intelligent, programmable, and environmentally sustainable optical security technologies.

## Methods

### Experimental setup

In this study, a customized FsL-3DP system based on TPP was employed for the fabrication of metastructures. The system primarily consists of a near-infrared femtosecond laser (ErFemto-780HP, central wavelength 780 nm, pulse width 150 fs, repetition rate 80 MHz), an XY-plane translation stage (ABL1500WB, Aerotech), a Z-axis translation stage (ANT130LZS, Aerotech), a scanning galvanometer (AGV10HP, Aerotech), and a high-numerical-aperture objective lens (60×/1.4 NA, Nikon). The coordinated movement of the translation stages and the scanning galvanometer is controlled by a custom-developed software platform, which converts complex 3D models into machine-readable fabrication instructions. During the printing process, small-area structures are rapidly fabricated using the scanning galvanometer alone, whereas large-area structures are completed through the synchronized motion of the scanning galvanometer and the translation stages, balancing both processing efficiency and fabrication precision.

### Fabrication of metastructures

An acrylate-based photoresist was drop-cast onto standard microscope cover glasses that had been sequentially cleaned with ethanol and deionized water and subsequently dried. The prepared samples were then mounted onto the fabrication system, where femtosecond laser 3D printing was performed under optimized parameters with a laser power ranging from 15 to 20 mW. After the fabrication of the structures, the samples were immersed in a propylene glycol monomethyl ether acetate (PGMEA) solution for 5 min to fully remove the unpolymerized photoresist. Subsequently, the samples were transferred to the chamber of a critical point dryer (Autosamdri-815, Tousimis), which had been pre-filled with isopropanol to completely submerge the samples. This step effectively prevented structural collapse caused by capillary forces during the drying process, thus preserving the integrity of the micro- and nanostructures.

### Simulation

The transmission spectra were simulated using the finite-difference time-domain (FDTD) method. A metastructure unit consisting of 2 × 2 nanopillars was constructed on a glass substrate. A broadband plane wave source was placed beneath the substrate and illuminated the sample perpendicularly (normal incidence). A monitor plane was positioned above the metastructures to record the far-field transmitted light intensity, thereby obtaining the transmission spectra. The collection ability of objective lenses with different NA was simulated by adjusting the collection range of the monitor.

### Morphological characterization

Optical images of the fabricated structural color samples were captured using a transmission optical microscope (CX31, Olympus). Additionally, a reflection optical microscope (LW200-3JT, CEWEI), a dark-field optical microscope (L608-HK810, AOSVI), and a laser confocal microscope (FV1200, Olympus) were employed to evaluate the security of the structural color encryption. The surface morphology of the samples was observed using a scanning electron microscope (SEM, JSM-7500F, JEOL). To prevent damage to the structures caused by high-energy electron beams, SEM imaging was performed under an accelerating voltage of 2 kV and a probe current of 10 μA.

### Optical properties characterization

The transmission spectra of the structural colors were measured using a custom-built micro-area transmission testing system equipped with a high-sensitivity spectrometer (AvaSpec-2048, Avantes). During the dynamic color modulation experiments, a controlled amount of deionized water was first injected onto the surface of the structures using a commercially available disposable syringe, allowing the liquid to spontaneously spread and cover the structural area. Subsequently, glycerol solutions (Aladdin Chemical) with different volume fractions were introduced, where the diffusion and mixing of glycerol within the solution enabled precise modulation of the environmental refractive index surrounding the structures.

## Supplementary information

Supplementary Information

Fabrication process of metastructures

Time-programmable coloration

Information decryption

Information self-destructs

## Data Availability

The data that support the findings of this study are available from the corresponding author upon request. Source data are provided with this paper.

## Code availability

All the relevant code used to generate the results in this paper and Supplementary information is available upon request.
