# Supplementary material for: Time-programmable coloration via 3D metastructures for optical encryption
Source: Light Sci Appl. 2026 Feb 19;15:118. doi: 10.1038/s41377-026-02202-y (PMC12921281; doi:10.1038/s41377-026-02202-y)
Supplement: Supplementary file 1 — Supplementary Information [file 41377_2026_2202_MOESM1_ESM.docx]

**Supplementary Information**

**Time-Programmable Coloration via 3D Metastructures for Optical Encryption**

Ming-Ze Zhao^1^, Zhi-Yong Hu^1,2,^*, Yi-Han Tao^1^, Ze-Xin Zhou^1^, Li-Jun He^3^, Zhen-Nan Tian^1^, Xue-Qing Liu^1,^*, Qi-Dai Chen^1^, and Din Ping Tsai^2,^*


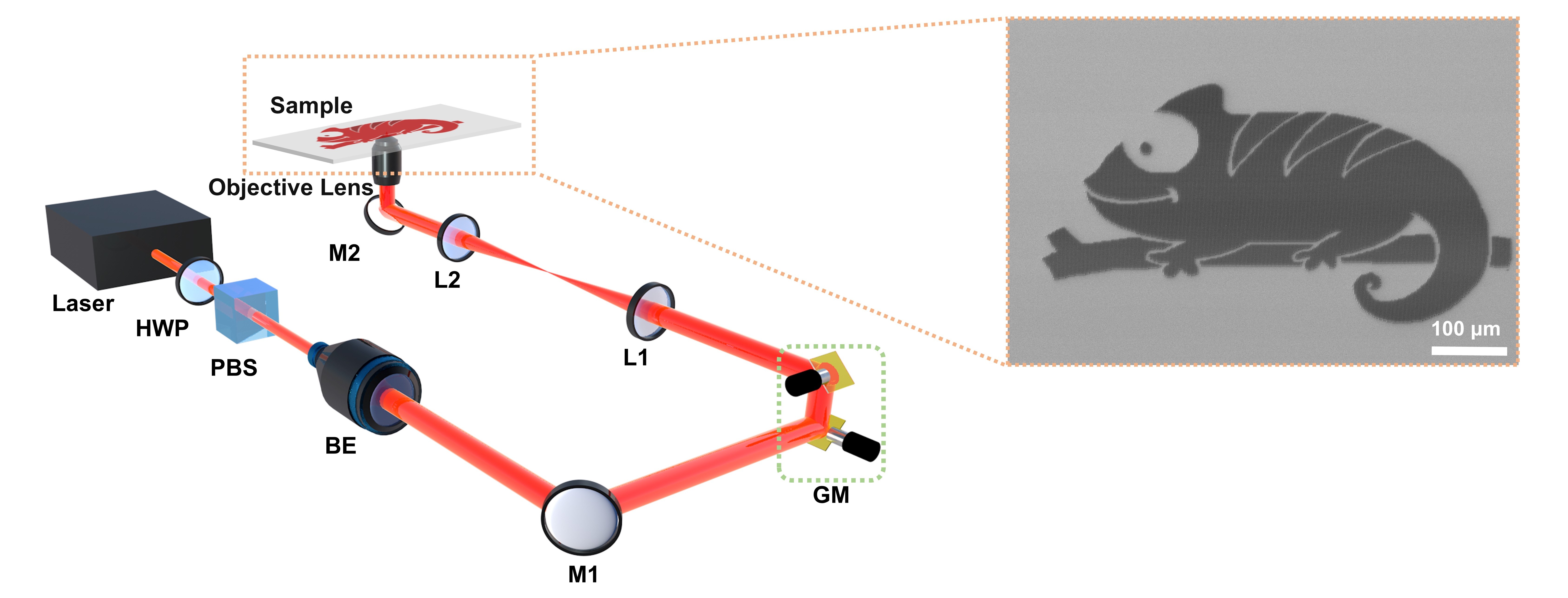


**Figure S1. Schematic illustration of femtosecond laser 3D printing for time-programmable structural colors.** HWP denotes half-wave plate; PBS, polarization beam splitter; BE, beam expander; M, mirror; GM, galvanometer scanner; and L, lens. The inset shows a scanning electron microscopy (SEM) image of the fabricated chameleon-patterned 3D metastructures (30° tilted view).

The **Figure S1** illustrates the experimental optical setup for fabricating three-dimensional metastructured nanostructures via femtosecond laser direct writing. Linearly polarized femtosecond pulses emitted from the laser are first adjusted in polarization state and power by a half-wave plate and a polarization beam splitter, followed by a beam expander for spot-size enlargement and collimation. The beam is then directed by mirror M1 and guided through a galvanometer scanner before entering a 4f system composed of lenses L1 and L2, which ensures strict mapping of the scanning plane and thereby maintains high-precision correspondence between the focal position and scanning displacement. After reflection by mirror M2, the pulses are tightly focused into the sample by a high-numerical-aperture objective lens, enabling high-quality fabrication of three-dimensional metastructured nanostructures. This optical design achieves high spatial resolution while allowing rapid scanning over large areas, thereby meeting the requirements for precise fabrication of complex meta-patterns.


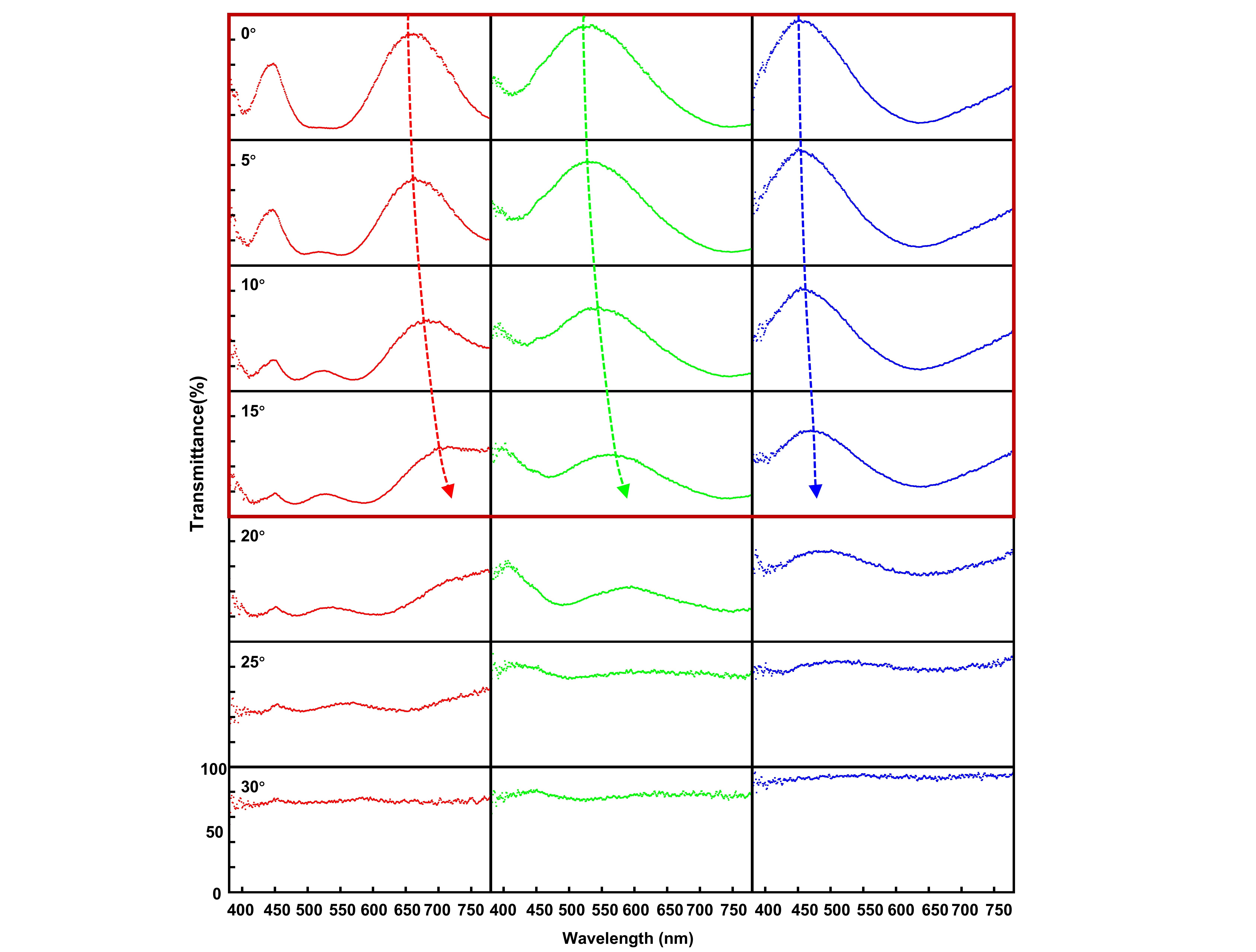


**Figure S2 Simulated transmittance of RGB meta-structures under varying NA conditions.**


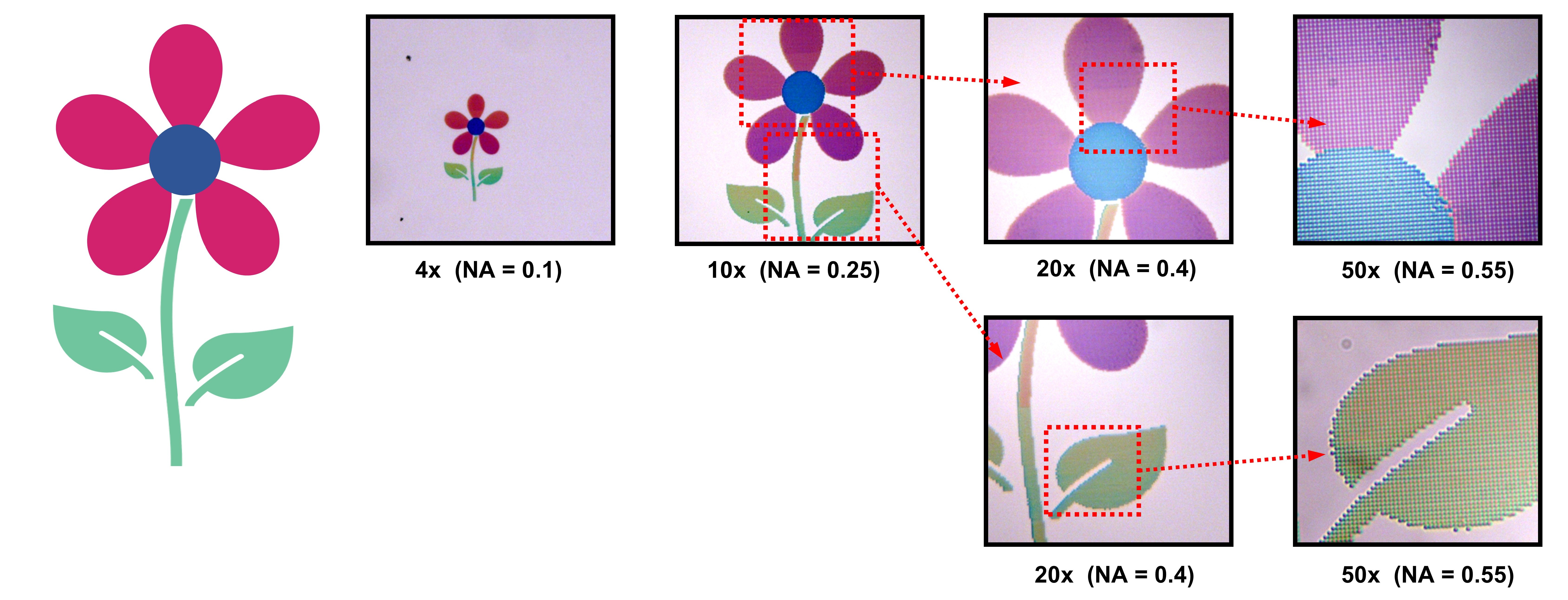


**Figure S3 Schematic of structural color pattern (left) and optical microscopy images of the structural color pattern captured using objective lenses with different NA (right).**


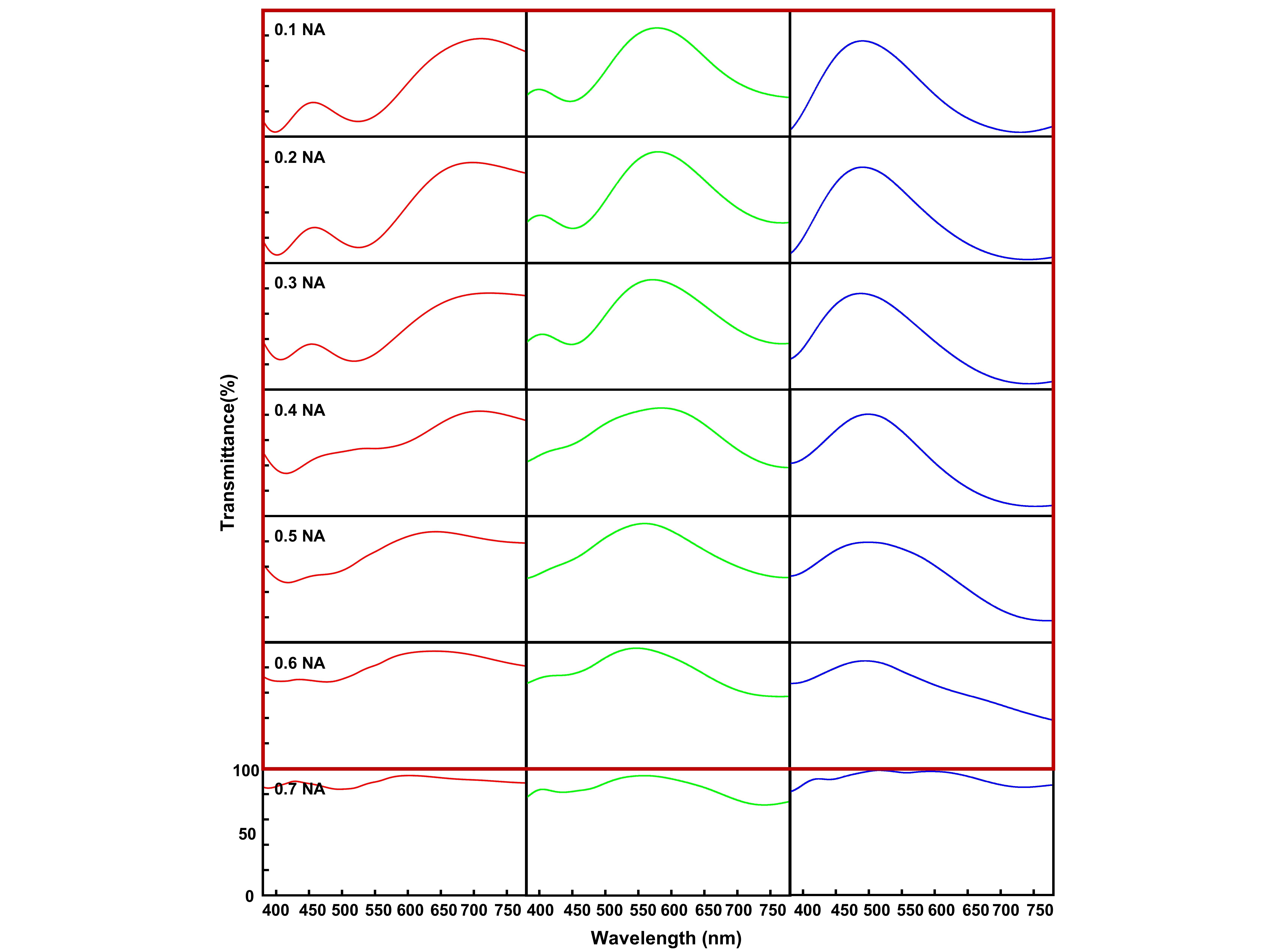


**Figure S4 Angle-resolved spectra of RGB meta-structures.**


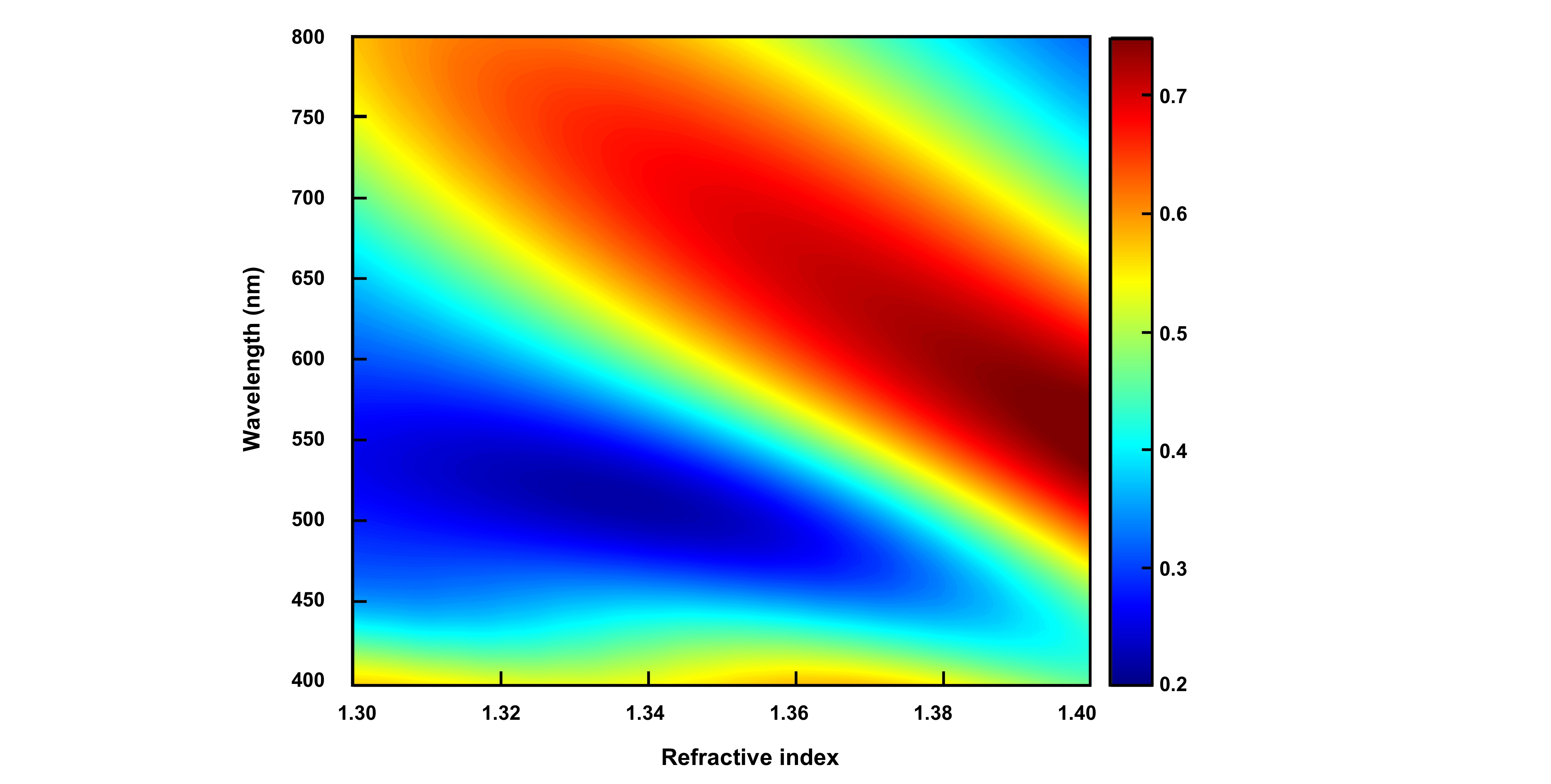


**Figure S5 Simulated transmittance of the same structure under different ambient refractive indices.**


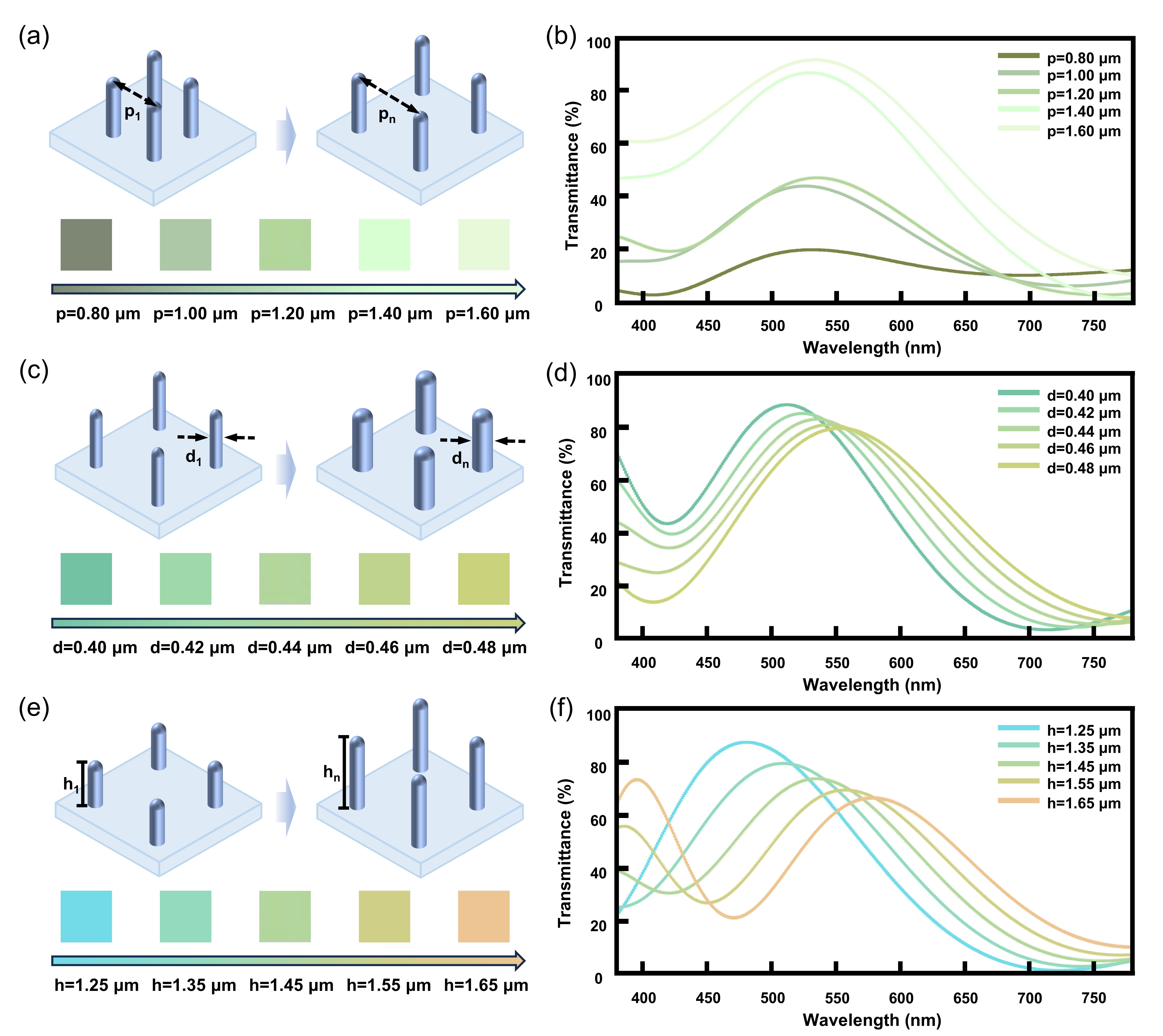


**Figure S6 Effect of metastructure geometric parameters on coloration.** **(a)** With the diameter fixed at 0.44 μm and the height at 1.45 μm, color variations of the metastructures at different periods. **(b)** The corresponding transmission spectra. As the period increases, the spectral peak intensity rises while the peak position remains nearly unchanged, indicating that the period primarily affects the transmittance and thus the brightness, with little impact on hue. **(c)** With the period fixed at 1.20 μm and the height at 1.45 μm, color variations at different diameters. As the diameter increases, the peak intensity decreases slightly and the peak exhibits a small red shift. Overall, the diameter modestly affects both hue and brightness. **(d)** The corresponding transmission spectra. **(e)** With the period fixed at 1.20 μm and the diameter at 0.44 μm, color variations at different heights; **(f)** the corresponding transmission spectra. As the height increases, the peak intensity decreases slightly while the peak undergoes a pronounced red shift, indicating that height shows the strongest correlation with the resulting color.


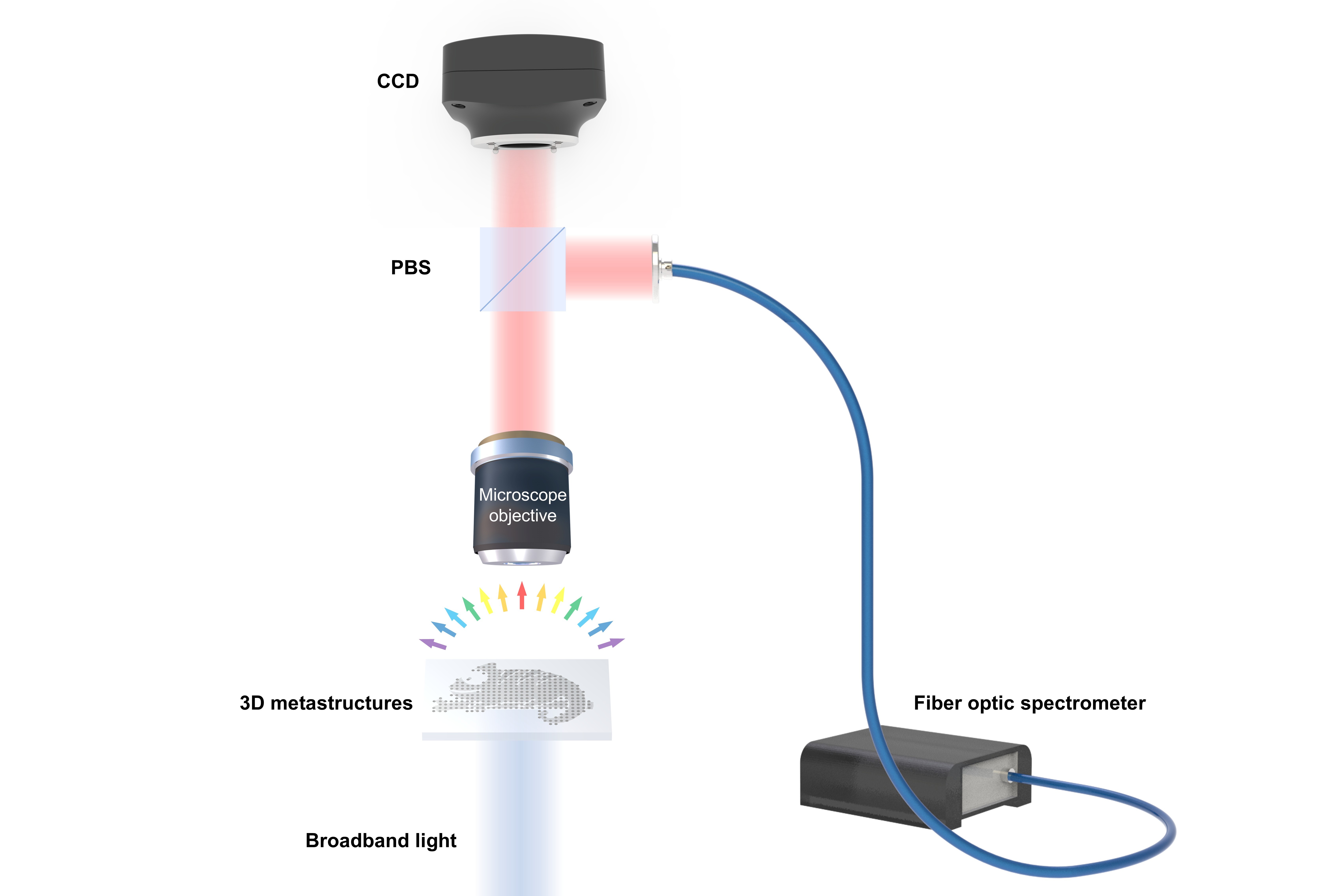


**Figure S7 Schematic of the far-field measurement platform.**


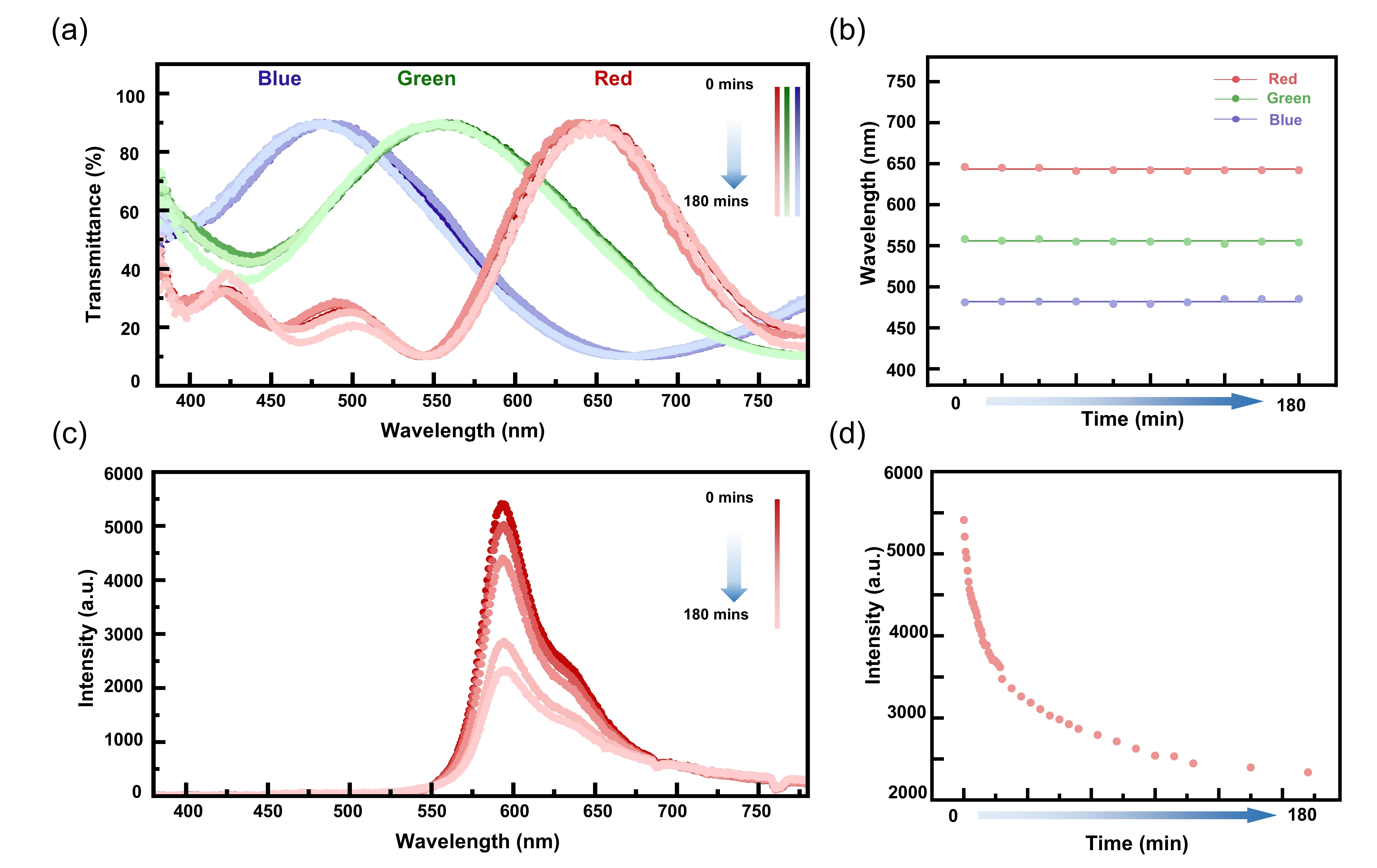


**Figure S8 Comparative experiment on the photostability of structural colors. (a)** Transmittance spectra of RGB micro/nanostructures under UV exposure; color from dark to light (five levels) indicates the progression of exposure time. **(b)** Statistical analysis of the peak position shifts in the transmittance spectra shown in (a). **(c)** Fluorescence emission spectra of Rhodamine B under UV exposure; color from dark to light (five levels) indicates the progression of exposure time. **(d)** Statistical analysis of the peak position shifts in the fluorescence emission spectra shown in (c).


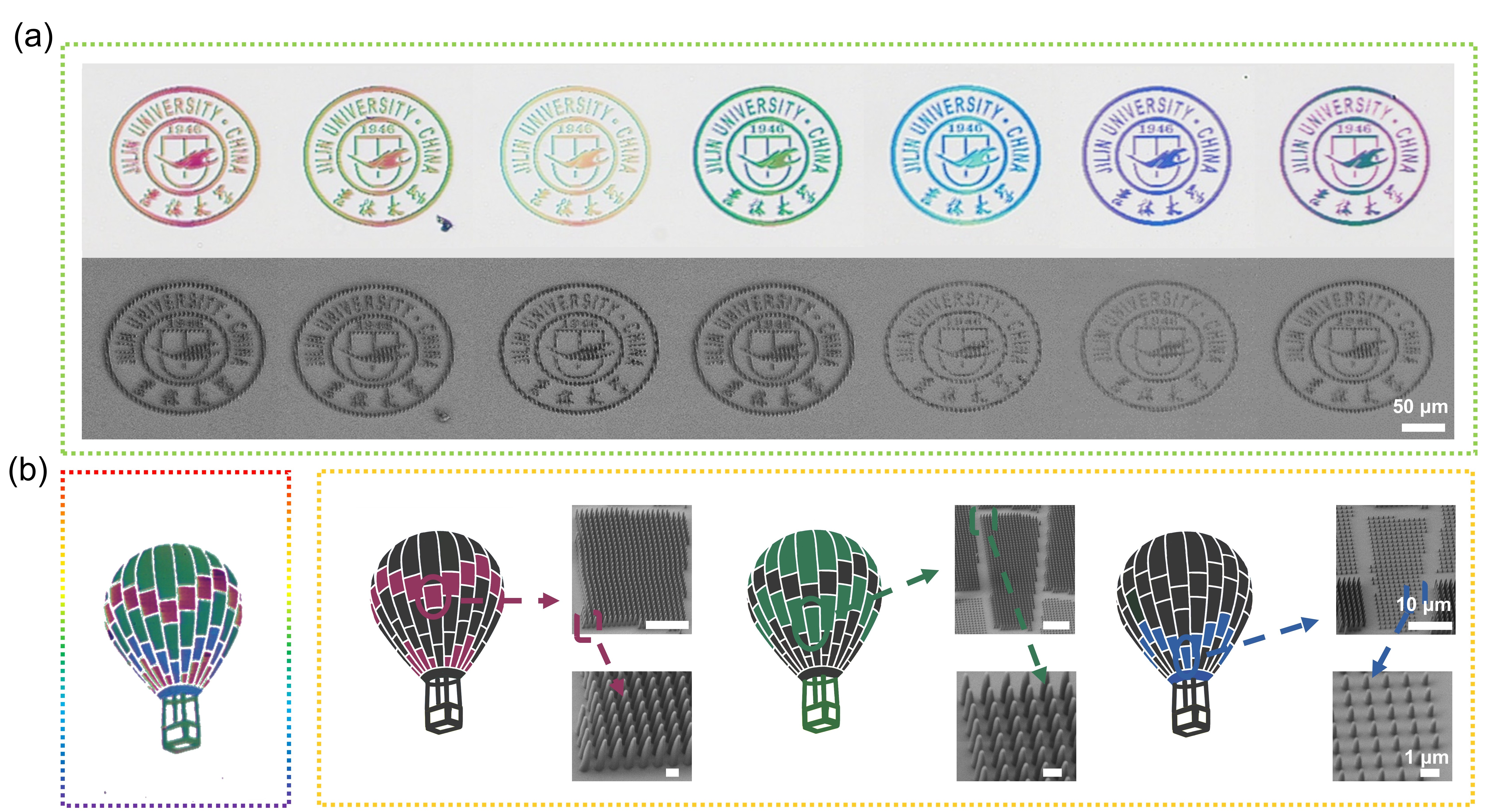


**Figure S9 Patterned printing of structural colors. (a)** Optical microscopy and SEM images of a monochromatic Jilin University emblem pattern. **(b)** Optical microscopy image of a hot air balloon pattern composed of RGB colors (left); schematic of the monochromatic segments within the pattern (right), with insets showing magnified SEM images of the corresponding monochromatic regions.


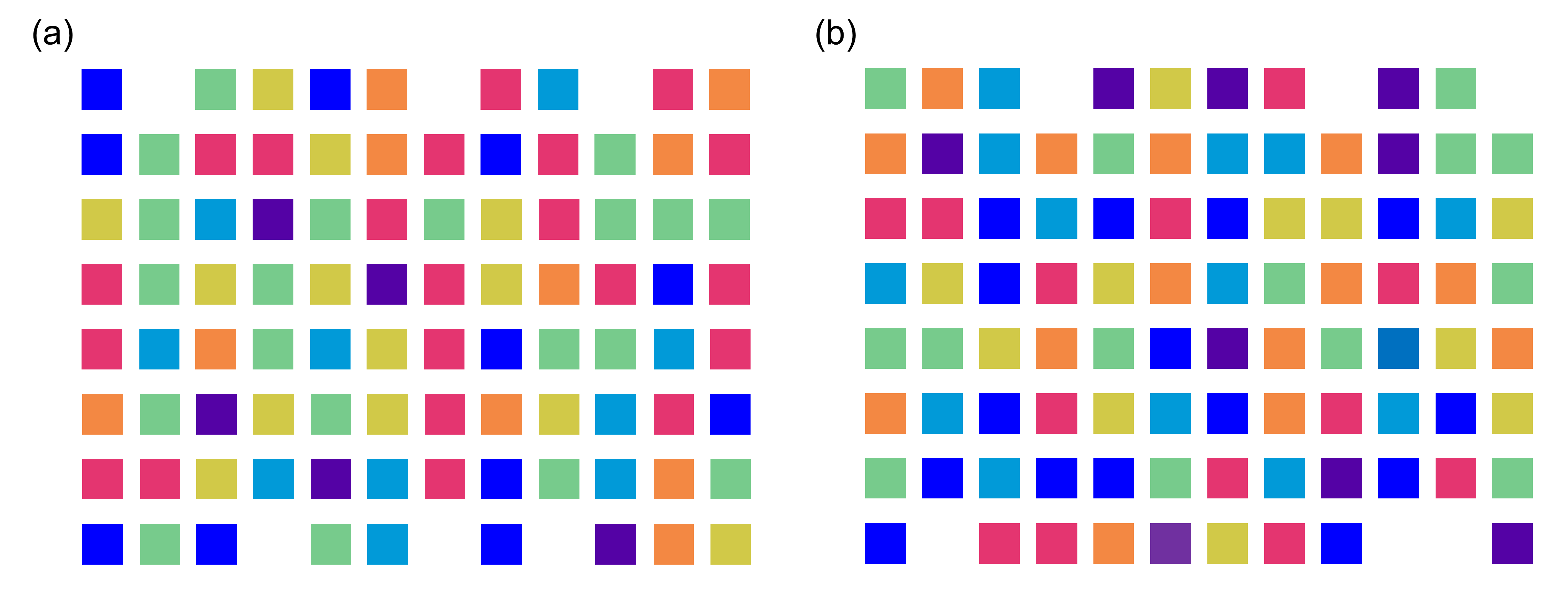


**Figure S10 Schematics of two structural color anti-counterfeiting label templates. (a)** Schematic of label 1 template. **(b)** Schematic of label 2 template.


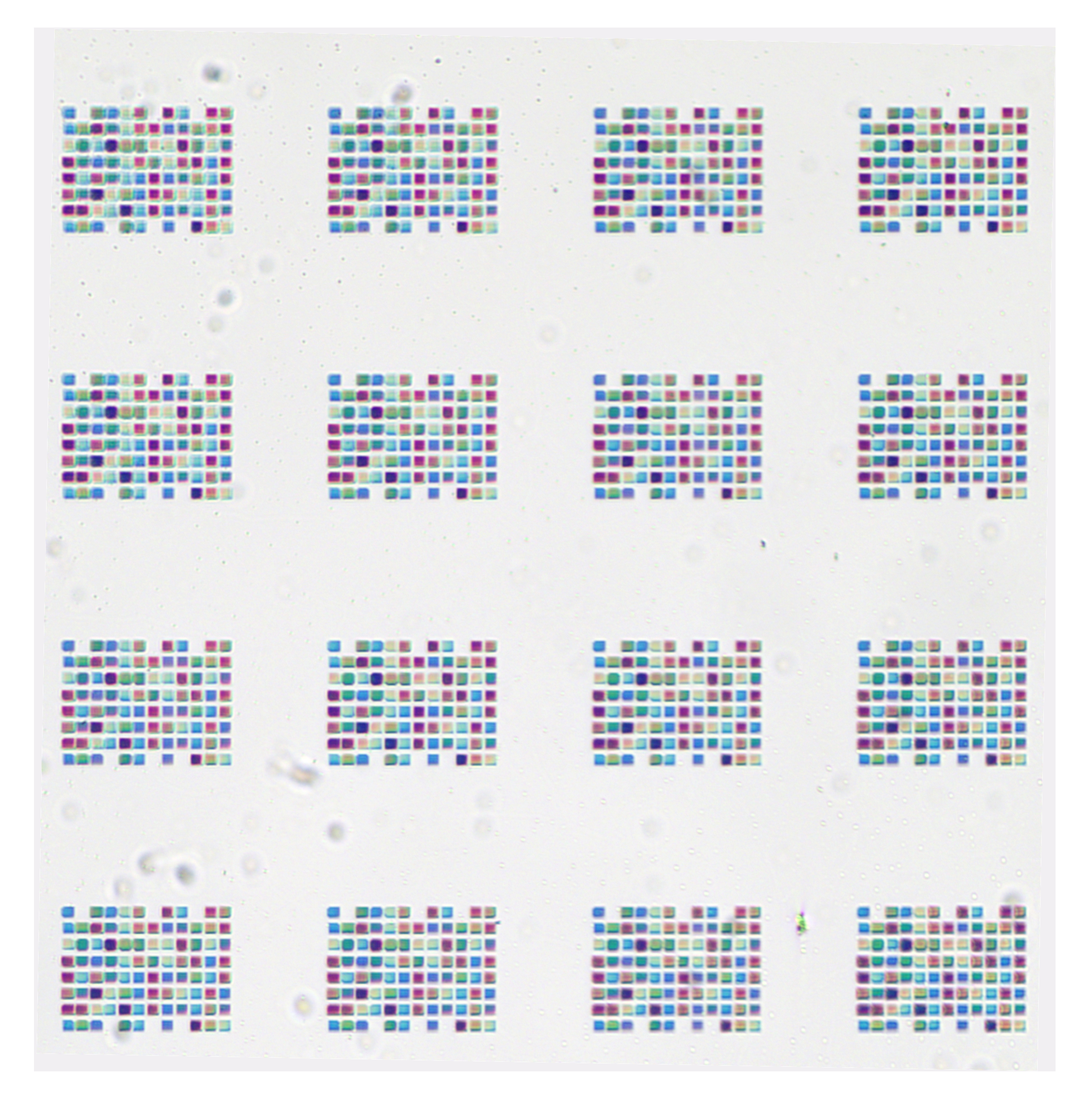


**Figure S11 Optical microscopy images of anti-counterfeiting labels printed in batch production.**


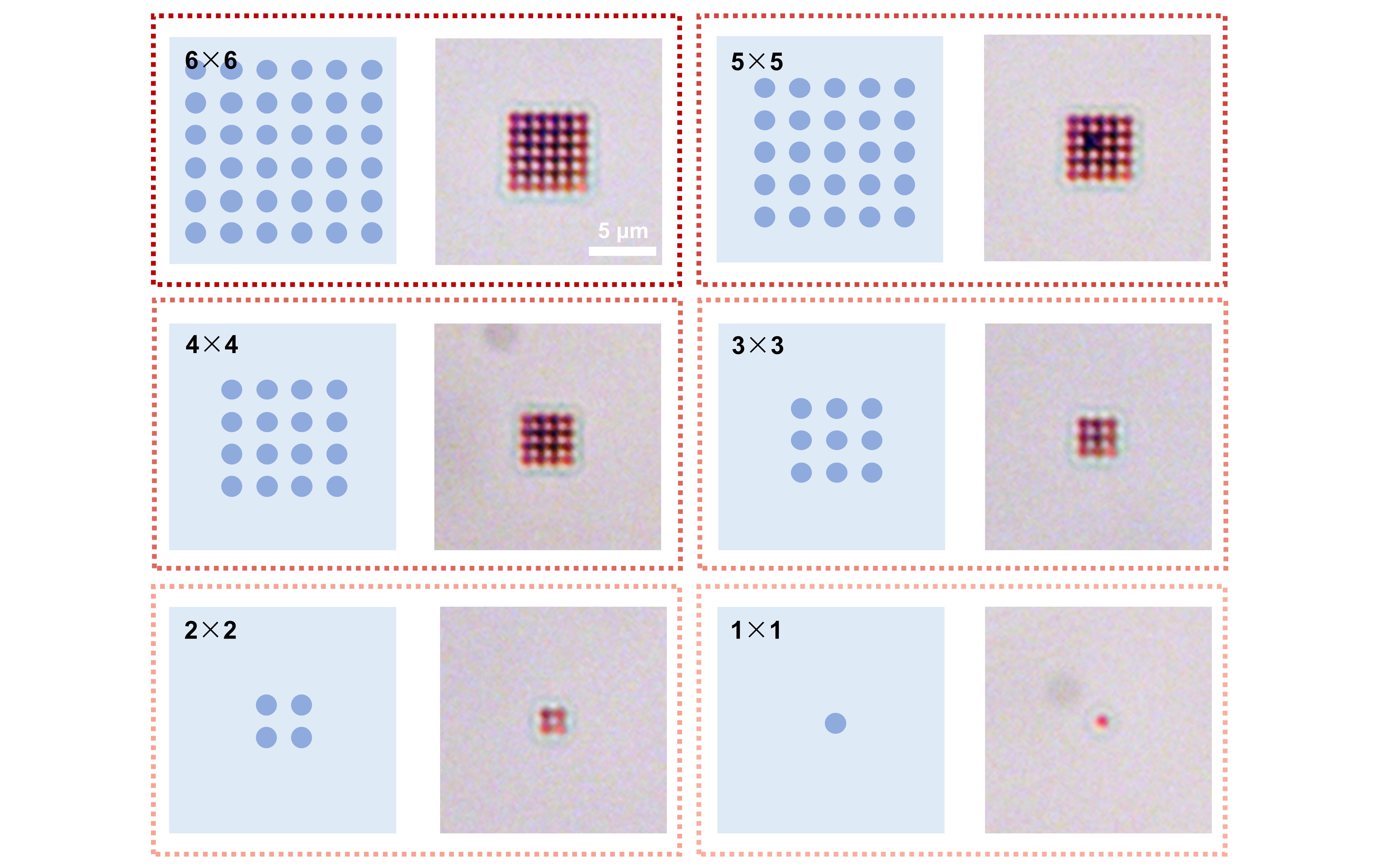


**Figure S12 Schematic diagram of metastructures at different scales and the corresponding microscope images.**


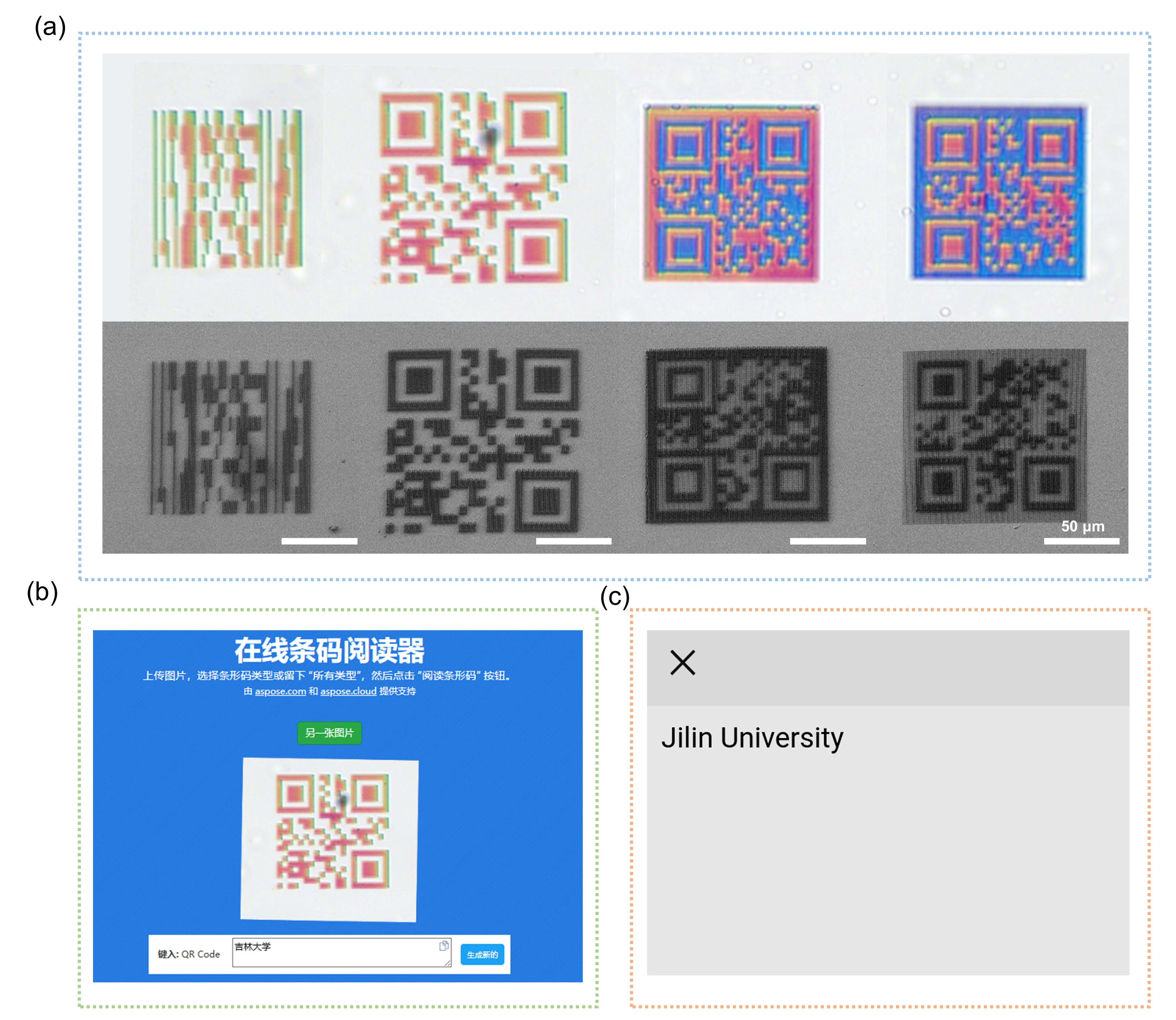


**Figure S13 Information storage and retrieval based on microscale barcodes/QR codes. (a)** Optical microscopy (top) and SEM (bottom) images of printed barcodes (far left), monochromatic QR codes (second from left), and dual-color QR codes (right two). **(b)** Decoding result of the monochromatic QR code. **(c)** Decoding result of the dual-color QR code.


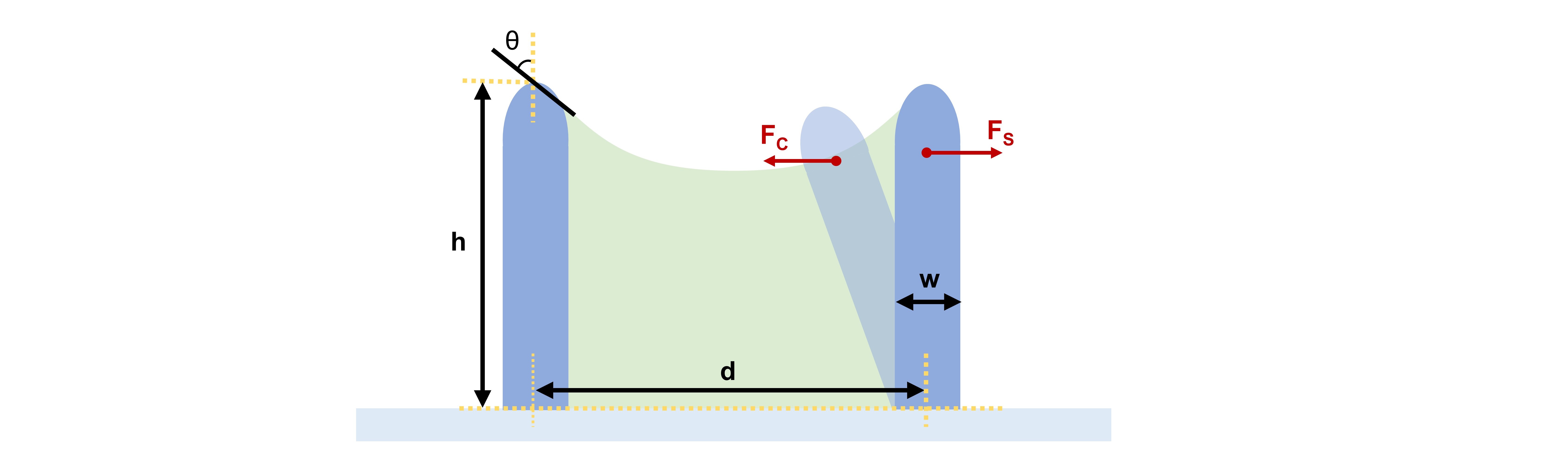


**Figure S14 Schematic illustration of stress experienced by the superstructure during liquid evaporation.**


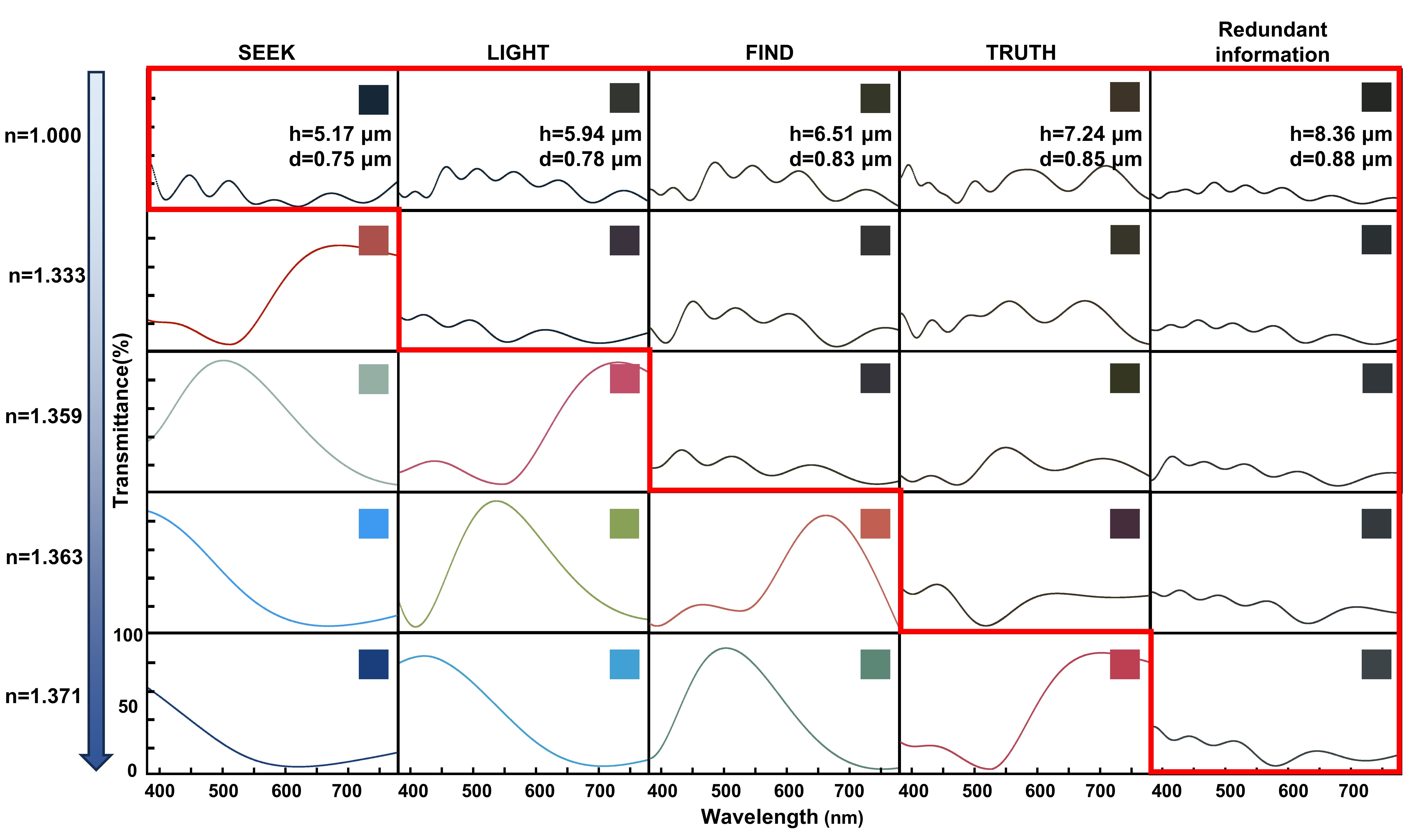


**Figure S15 The simulated transmission** **spectra of the metastructures corresponding to different information layers under a gradient refractive index.** The insets show the corresponding perceived colors. All structures share a period of 1.6 μm, and the labels below each inset denote the structure height h and diameter d. The spectra within the red box are in the encrypted state, while those outside the box represent the spectra of the metastructures after decryption.

The coloration of a metastructure is governed not only by its geometry but also by the refractive index of the surrounding environment. At lower environmental refractive indices, the metastructure supports more pronounced resonances and thus exhibits distinct colors; as the environmental index increases, the index contrast between the structure and its surroundings diminishes, which modifies the resonant modes and, in turn, the displayed color. In particular, when the structure height exceeds a certain threshold, the transmittance is suppressed across the entire visible band and the metastructure appears black. This implies that, in a high index-contrast environment, taller structures tend to appear black even when the nanocolumn geometries differ.

Leveraging this behavior, we simulated the transmission spectra of metastructures with different geometrical parameters under various environmental refractive indices and selected appropriate designs to construct distinct information layers. As shown in **Figure S15**, when the metastructures are in air, all designs appear black. With increasing environmental refractive index, the index contrast gradually decreases, at which point the low-height structures in the first information layer undergo a change in resonant mode and transition from black to red, marking the decryption of the first layer. The remaining metastructures remain black, indicating that they are not yet decrypted. Further increases in the environmental index sequentially decrypt the second through the final layers, while the redundant elements remain black throughout, thereby achieving time-sequenced decryption.


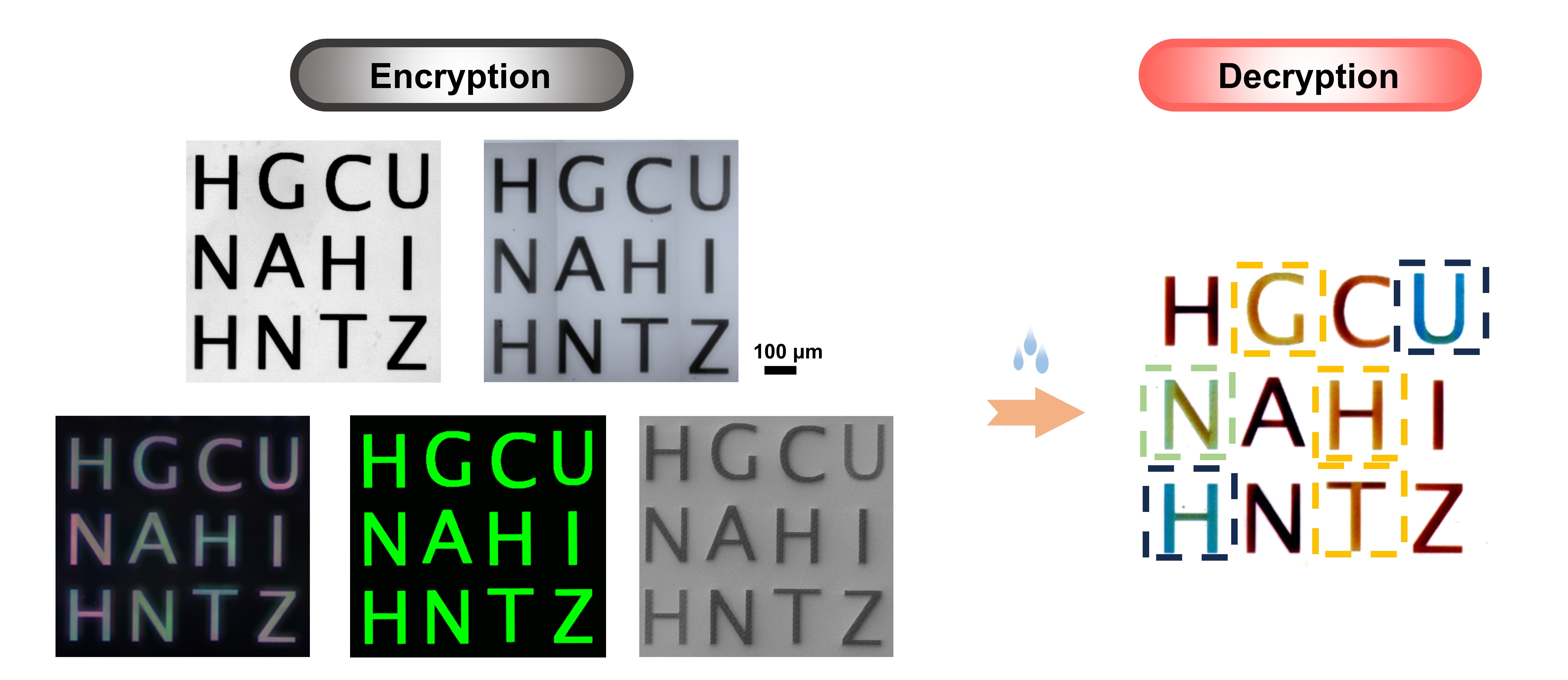


**Figure S16 Local comparison of the random alphabet in encrypted and decrypted states.** The left panel shows the encrypted state, including optical microscopy images in transmission (top left) and reflection (top right) modes, dark-field microscopy (bottom left), fluorescence microscopy (bottom center), and SEM (bottom right) images. The right panel shows the decrypted state, represented by a transmission optical microscopy image of a localized region of the random alphabet (magnified from **Figure 4c** in the main text).
